# Designing antibodies against LRRK2-targeted tau epitopes

**DOI:** 10.1371/journal.pone.0204367

**Published:** 2018-09-27

**Authors:** Matthew Hamm, Thomas B. Ladd, Yona Levites, Todd E. Golde, Benoit I. Giasson, Jada Lewis

**Affiliations:** 1 Department of Neuroscience, College of Medicine, University of Florida, Gainesville, Florida, United States of America; 2 Center for Translational Research in Neurodegenerative Disease, College of Medicine University of Florida, Gainesville, Florida, United States of America; 3 McKnight Brain Institute, College of Medicine University of Florida, Gainesville, Florida, United States of America; McGill University, CANADA

## Abstract

Phosphorylation of the microtubule associated protein tau is an important modulator of its normal physiological functioning; however, it may also contribute to tau mis-folding and aggregation in neurodegenerative diseases, which are collectively termed tauopathies. As such, the investigations of tau phosphorylation and kinases that modify tau are important in trying to elucidate tau function and the mechanisms involved in the development of tauopathies. We have recently demonstrated that the putative tau kinase leucine-rich repeat kinase 2 is capable of phosphorylating tau at threonines 169 and 175 *in vitro*, and it has been previously shown that hyperphosphorylation at threonine 175 occurs in filamentous tau species from Alzheimer’s brain tissue. These prior findings suggest that further studies of phosphorylation of tau at these epitopes may shed light on the pathogenesis of tauopathies. There is, however, a lack of tools available to analyze phosphorylation of tau at these sites. This study aimed to bridge that resource gap by generating monoclonal antibodies against tau phosphorylated at either threonine 169 or 175. While we did not succeed in generating a phospho-specific antibody, we did generate an antibody, MHT2, which is specific for human tau encompassing the threonine 169/175 epitope region. Immunostaining of transgenic rTg4510 mouse tissue as well as human tauopathy cases with MHT2 indicates that this antibody selectively detects cytoplasmic tau in the form of neurofibrillary tangles, and that it may have a further specificity pertaining to severity of disease progression, either because of phosphorylation or conformational bias.

## Introduction

The hyperphosphorylation and aggregation of microtubule associated protein tau is a pathological hallmark of a wide swath of neurodegenerative diseases including Alzheimer’s disease (AD), progressive supranuclear palsy (PSP), and frontotemporal dementia (FTD), which are collectively termed tauopathies. Site-specific hyperphosphorylation of tau is concomitant with the aberrant aggregation and accumulation of tau [1], making the study of tau kinases and tau phosphorylation an important point of interest in researching tauopathies. Our labs and others have recently demonstrated that leucine-rich repeat kinase 2 (LRRK2) can be a tau kinase and an activator of other tau kinases including glycogen synthase kinase (GSK)-3β [2–7].

In our previous findings, *in vitro* incubation of tau with G2019S LRRK2 resulted in phosphorylation of tau at several sites including threonines 149 (T149), 153 (T153), 169 (T169), and 175 (T175) [3]. We also demonstrated phosphorylation of T149 and T153 in human cases of tauopathy. Additionally, an increase in phosphorylation of T149 was detected in the detergent insoluble fraction of brain tissue taken from rTg4510 mice that overexpressed wild-type human LRRK2 as compared to rTg4510 tissue. While this previous data supports a role for LRRK2 in tau phosphorylation, we have not yet further explored T169 and T175 in this context, and those sites are also relatively little explored in the tau field in general. Hyperphosphorylation at T175, but not T169, has been detected in paired helical filament (PHF) tau from AD brain tissue [8]. T175 phosphorylation has been linked to fibril formation and cell death, and has been detected via immunohistochemistry in an array of tauopathies including AD and amyotrophic lateral sclerosis [9,10]. While an antibody directed against phosphorylated T175 was designed and used in these immunohistochemical studies, there are no commercially available antibodies targeting tau T169 or T175, phosphorylated or not, representing a gap in available resources to study modifications of tau at these particular sites of interest.

In our current study, we sought to expand the antibody reagents available to study T169 and T175 of tau in normal physiology and disease. However, we generated an antibody that is specific for the region of tau encompassing T169 and T175. Importantly, this region bears several amino acid differences between the mouse and human tau proteins. The resultant tau antibody, termed MHT2 here, specifically recognizes human tau. Interestingly, it proved effective in immunostaining late-stage tauopathy in both human disease cases as well as a mouse model of tauopathy. This may suggest the ability of the antibody to immunostain late tauopathy reflects the changing conformation of tau and thus epitope presentation as tauopathy progresses.

## Materials and methods

### Monoclonal antibody production

The peptides 163-179(pT169) and 163-179(pT175) corresponding to amino acids 163–179 in human tau with either T169 or T175 phosphorylated, respectively, and an added C residue at the carboxyl-terminus (see Table 1), were synthesized and purified by GenScript (Piscataway, NJ). After reconstitution in PBS and conjugation to Imject maleimide-activated mariculture keyhole limpet hemocyanin (mcKLH; Thermo Scientific, Waltham, MA), the peptides were used as antigens for monoclonal antibody production performed as previously described [11].

**Table 1:** Peptides used for antibody production and characterization. Peptides are named according to amino acid positions in human 2N/4R tau. [p] = phosphorylated.

Peptide name	Sequence	Corresponding amino acids in tau
**146–159** (pT149)	DGK[p]TKIATPRGAAC	146–159
**146–159** (pT153)	DGKTKIA[p]TPRGAAC	146–159
**146–159** (pT149 &153)	DGK[p]TKIA[p]TPRGAAC	146–159
**146–159**	DGKTKIATPRGAAC	146–159
**165–176** (pT169)	QANA[p]TRIPAKTPC	165–176
**165–176**	QANATRIPAKTPC	165–176
**163–176** (pT169)	KGQANA[p]TRIPAKTPC	163–176
**163–176**	KGQANATRIPAKTPC	163–176
**163–179** (pT169)	KGQANA[p]TRIPAKTPPAPC	163–179
**163–179** (pT175)	KGQANATRIPAK[p]TPPAPC	163–179
**163–179**	KGQANATRIPAKTPPAPC	163–179

### Hybridoma screening

All hybridoma clones were screened for reactivity to tau by enzyme-linked immunosorbent assay (ELISA). MaxiSorp plates (Thermo Scientific, Waltham, MA) were coated with 100 ng of a tau-based peptide or 500 ng recombinant human tau in PBS overnight at 4°C and blocked with 5% FBS/PBS the following day for 1 hour. Hybridoma media was then applied to plates and incubated at room temperature for 3 hours. Following this incubation, the plates were washed several times with PBS, and the incubated with goat anti-mouse secondary antibody conjugated to horse radish peroxidase (HRP; Jackson Immuno Research Labs, West Grove, PA) for 1 hour at room temperature. After another round of thorough PBS washes, TMB substrate was applied to the plates until a color change was observed (Pierce, Rockford, IL). Reactions were quenched with 1M HCl and absorbance was measured at 450 nm.

### Other antibodies

Other tau antibodies used herein include total tau polyclonal antibody 3026 [11], and tau monoclonal antibody PHF1 (specific for pS396/S404), provided by Dr. Peter Davies, The Feinstein Institute for Medical Research, Manhasset, NY, USA. Anti-GAPDH monoclonal antibody GA1R was obtained from Thermo Fisher Scientific.

### Expression plasmid and tau recombinant proteins

The mammalian expression vector pcDNA3.1(+) expressing the full-length 2N4R human tau isoform was previously described [12]. Human 0N3R and 2N4R full-length tau cloned into the bacterial expression vector pRK172 was kindly provided by Dr. Michel Goedert. The pRK172 plasmid expressing C-terminal fragment of human 3R tau [C′ Tau] corresponding to amino acids 244–441 minus amino acids 275–305 that would be present in 2N4R tau was previously described [6]. Expression of the pRK172 tau construct in *E*. *coli* BL21 and subsequent purification of recombinant tau proteins has been previously described [13].

### Cell culture and transfection

HEK293T cells (ATCC) were maintained with DMEM supplemented with 10% FBS, 100 units penicillin/ml, and 100 μg streptomycin/ml, at 37 °C and 5% CO_2_. The cells were plated on polystyrene 6-well plates. At ~30–50% confluency, cells were transfected with the mammalian expression vector pcDNA3.1(+) containing cDNA for 2N4R human tau using calcium phosphate precipitation. Empty pcDNA3.1(+) vector was used as a negative control. For transfection per 2 ml of cell culture media, 3 μg of plasmid DNA was diluted into 37.5 μl of 250 mM CaCl_2_ and stepwise added to an equal volume of 50 mM *N*,*N-bis*(2-hydroxymethyl)-2-aminoethanesulfonic acid, 280 mM NaCl, 1.5 mM Na_2_HPO_4_, pH 6.96. This mixture was incubated at room temperature for 20 min before being added dropwise to the media in each well. After 48 hours, the cells were harvested and lysed in 3% SDS/50 mM Tris—HCl, pH 6.8 and heated at 100 °C for 5 min. Protein concentration was determined using BCA protein assay reagent and bovine serum albumin as the protein standard (Pierce,Fisher Scientific).

### Transgenic mice

The rTg4510 mouse model was sourced from the lab of Dr. Jada Lewis, N = 17. These mice overexpress human P301L tau in hippocampal and forebrain neurons, and have been previously described in detail [14,15]. The Tau-knockout mice were from Jackson Labs (Stock 007251), N = 2. Tau knockout mice lack endogenously expressed murine tau, and have also been characterized in detail elsewhere [16]. All procedures were performed according to the NIH Guide for the Care and Use of Experimental Animals and were approved by the University of Florida Institutional Animal Care and Use Committee. Mice were maintained in a pathogen-free facility on a 12 h light/dark cycle with water and food provided ad libitum. Animals were group-housed to minimize stress in the laboratory setting.

### Tissue harvest and preparation

Mice were euthanized via cervical dislocation so as to best preserve phospho-moieties of tau. Brain tissue was immediately harvested and processed for immunohistochemistry and western blotting as previously described [3].

### Human brain tissue

Formalin fixed, paraffin embedded human brain tissue from de-identified donors was obtained from the University of Florida Neuromedicine Human Brain Tissue Bank.

### Enzyme-linked immuno sorbent assay (ELISA) for assessment of antibody specificity

This method was previously described in detail [3]. ELISA screens were performed to test specificity of antibodies MHT1 and MHT2, using PHF1 antibody as a control. Different polypeptides representing regions of tau with and without epitope-specific phosphorylation were used as targets in the screening. The full list of tau-based peptides used in these ELISAs is shown in Table 1. In addition to these peptides, a 0N3R tau C-terminal fragment was used as a further control, both under non-phosphorylated and GSK-3β-phosphorylated conditions. Finally, a 2N4R full-length recombinant human tau protein was also used. ELISA data was organized and analyzed using Prism GraphPad, V6 (http://www.graphpad.com/scientific-software/prism/).

### Western blotting

Western blot analyses were used to assess tau-transfected HEK293T lysate and murine tissue homogenates. Hemi-brain tissue homogenates from nontransgenic, tau-knockout, and rTg4510 mice were fractionated into soluble and detergent insoluble fractions as previously described [15]. For each immunoblot, equal amounts of samples were loaded onto each lane and then resolved on SDS-PAGE gels. After SDS-PAGE gel resolution, samples were electrophoretically transferred to polyvinylidene difluoride (PVDF) membranes. PVDF membranes were blocked in Tris-buffered saline (TBS) with 5% dry-milk powder. After 1 hour in TBS/milk blocking solution, membranes were incubated with primary antibody at 4° overnight. Antibodies MHT1, MHT2, and PHF1 were diluted in TBS/5% BSA, while 3026 and GAPDH were diluted in TBS/5% milk. The next day, membranes were washed in TBS and then incubated for one hour in goat anti-mouse conjugated horseradish peroxidase (HRP) (Jackson ImmunoResearch, West Grove, PA) or goat anti-rabbit HRP (Jackson ImmunoResearch, West Grove, PA). To visualize immunoreactivity, each membrane was exposed with western lighting plus-ECL solution (PerkinElmer, Waltham, MA) and subsequently analyzed with a syngene PXi system. Following the original exposures, membranes were stripped for further immunoprobes. Membranes were incubated in stripping solution (5% SDS, 62.4 mM Tris-Base, pH 6.8, 0.7% 2-mercaptoethanol) for 15 minutes at 55°C, washed in TBS for 30 minutes, and then again processed for immunoblotting as described here.

### Immunohistochemistry

Fixed mouse brains were paraffin embedded and cut into 5 μm sagittal sections. Hematoxylin and eosin (H&E) staining was performed on at least two brain sections from each mouse to align all brains to approximately 0.7 mm lateral to the midline using a mouse brain atlas. Microscope slide-mounted tissues were incubated in 2 xylene washes for 5 minutes apiece, followed by sequential rehydration washes in 100/95/70% alcohol and finally H_2_O for 3 minutes apiece. Following rehydration, slides were boiled in 10 mM citrate buffer, pH 6.0, with 0.05% Tween-20 for 30 minutes. Slides were then rinsed in tapwater and phosphate-buffered saline (PBS) for 15 and 5 minutes, respectively. Peroxidase blocking was then performed by incubating the slides in 0.3% H_2_O_2_, 0.005% Triton-X in PBS for 20 minutes followed by another set of water and PBS rinses. After a final 5-minute rinse in PBS with 0.05% Tween-20 (PBS-T), slides were blocked with 10% normal goat serum in PBS-T for 30 minutes, prior to the application of primary antibody diluted in 5% normal goat serum in PBS-T. After an overnight incubation, slides were again rinsed in PBS-T for 5 minutes before a 30-minute incubation in goat-based secondary antibodies, again diluted in 5% normal goat serum/PBS-T. Following another PBS-T wash, slides were subjected to a 30 minute incubation in Avidin-Biotin Complex (ABC), diluted 1:1000 equal parts A and B in PBS-T. Finally, after a two 5-minute PBS wash, tissues were incubated in metal-enhanced DAB solution from ThermoFisher, prepared according to manufacturer guidelines. Following this chromagen reaction, slides were rinsed, treated with a hematoxylin counter-stain, dehydrated in an ascending ethanol/H_2_O series, washed in xylenes for 2x5 minutes, and coverslipped.

### Determination of equilibrium dissociation constant

BioLayer Interferometry (BLI) was used for KD (equilibrium dissociation constant) determination utilizing Streptavidin Biosensors (SA), on the Octet Red384 platform (Fortebio, Pall Life Sciences, CA,). MHT2 antibody was biotinylated with Pierce EZ-Link NHS-PEG4 (21455), Thermofisher, MA. Streptavidin Biosensors sensors were equilibrated and loaded to near-saturation with biotin-MHT2 in PBS, 0.002% Tween20 (assay buffer), transferred to fresh assay buffer for baseline measurement, then associated with recombinant 2N4R human tau along a serial dilution. The sensors were finally moved back to assay buffer for disassociation. Constant of association (ka), constant of dissociation (kd), and KD (kd/ ka) values were determined by global fitting of the binding curves for the ligand dilutions and calculated by applying a 1:1 interaction model using the ForteBio Data Analysis software version 9.0.0.14 (Fortebio, Pall Life Sciences, CA).

### mRNA isolation, cDNA synthesis, and amplification of cDNAs encoding variable regions

mRNA was isolated from subcloned MHT2 hybridomas cell lines using a mRNA isolation kit (Qiagen). cDNA was synthesized using MMLV Reverse Transcriptase (Promega) and random hexamers. The cDNA was then poly-G tailed with Terminal Transferase (New England BioLabs). After determination of isotype IgG1 using an isotyping kit (Sigma), cDNAs encoding the Variable heavy (VH) and Variable light (VL) chains were amplified using anchor PCR with a forward poly-C anchor primer and a reverse primer specific for constant region sequence of IgG1, as described in Gilliland, L.K., et al [17]. PCR products were than sequenced using the same primers and the consensus variable heavy and variable light chains were determined.

## Results

### Monoclonal antibody production against tau peptides phosphorylated at T169 and T175

**Fig 1:**
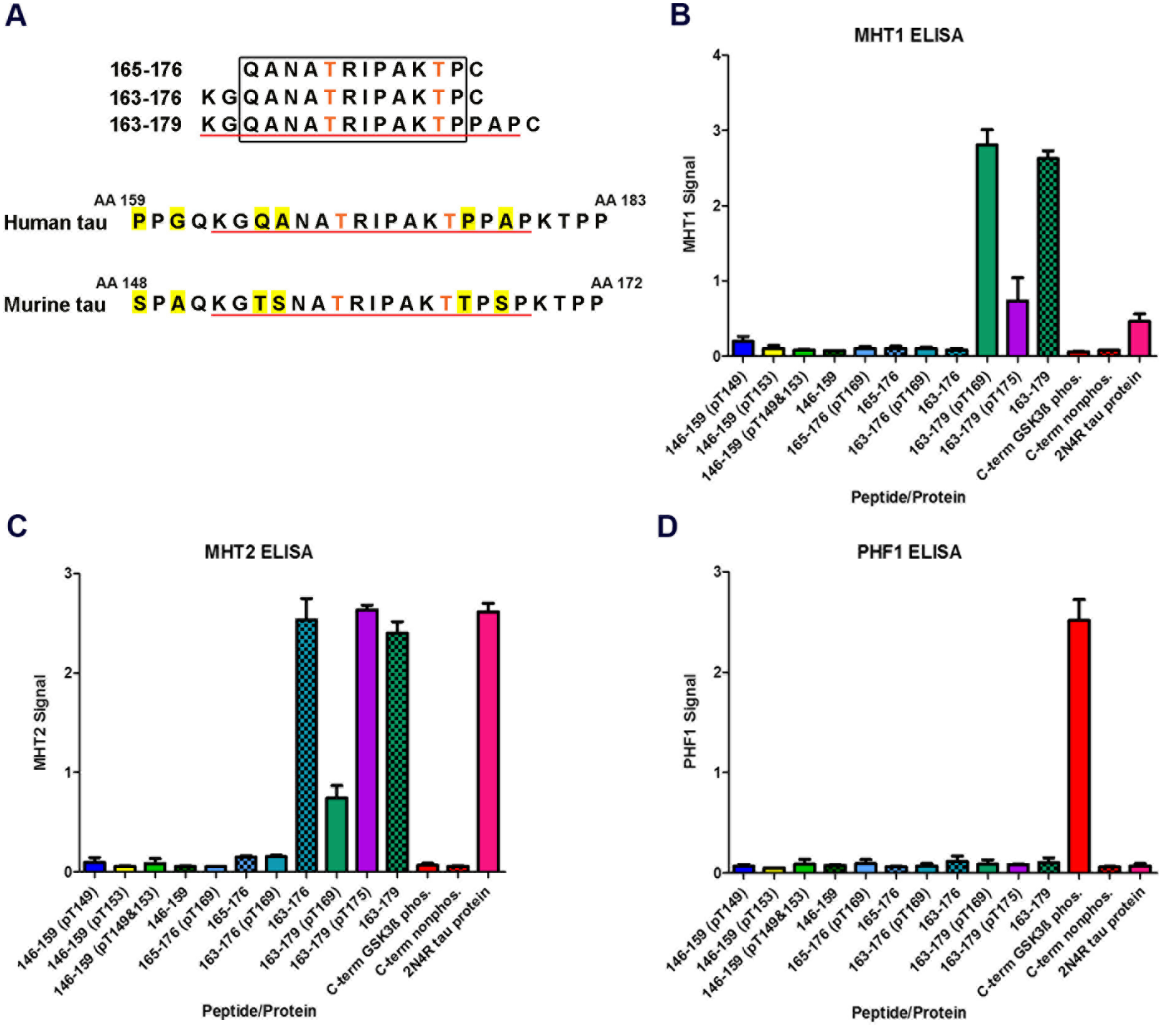
Characterization of the specificity of novel tau monoclonal antibodies by ELISA. (A) Sequences of the peptides used in attempts at generating tau monoclonal antibodies, as well as the corresponding amino acid sequences from full-length human and murine tau. T169 and T175 are represented with orange text. The overlapping region between the 163–179 peptide antigen and the actual tau sequence is underlined in red. Single amino acid differences between human and murine tau are highlighted in yellow. (B-D) ELISA data from experimental antibodies MHT1 and MHT2, as well as the well-characterized PHF1 antibody, respectively, as indicated above each graph. The ELISA plates contained triplicate sets of various phosphorylated peptides representative of different threonine epitopes on tau, as well as non-phosphorylated counterparts for each peptide. A truncated, C-terminal tau species (244–441) was also used as a control, with one batch of the C-terminal protein having been phosphorylated by GSK3β. Recombinant, full-length 2N4R tau protein was also used.

Our previous research indicates that LRRK2 phosphorylates tau *in vitro* and that the presence of microtubules enhance the ability of LRRK2 to phosphorylate tau at specific epitopes including T169 and T175 [3,6]. Having previously detected LRRK2-tau phosphorylation at T169 and T175 *in vitro* with mass spectrometry, we attempted to produce antibodies against these two phospho-epitopes so that they may be used as research tools in exploring LRRK2, tau, and related disease. We designed peptides (Table 1) that represent the segment of human tau between amino acids 163 and 179, containing both T169 and T175, with one or neither threonine site phosphorylated. Fig 1A demonstrates this region of interest, underlined in red, with differences between the murine and human full-length isoforms highlighted in yellow.

We attempted to produce monoclonal antibodies against the phosphorylated 169 and 175 threonine sites using a series of synthetic peptides that include either of the targeted phosphorylated residues (Table 1; Fig 1A). Antibody production against phosphorylated T169 was unsuccessful using either the short 165-176(pT169) or intermediate-length 163-176(pT169) peptide, and only upon further expanding the antigen sequence to the long 163-179(pT169) peptide, were we successful in generating hybridomas that actively produced what appeared to be site-specific antibodies. A similar attempt to produce antibody against phosphorylated T175 using the 163-179(pT175) peptide as an antigen was also successful in generating site-specific antibody. ELISA data for these results is shown in Fig 1.

Antibody “MHT1” (Fig 1B) was produced against peptide 163-179(pT169), while antibody “MHT2” (Fig 1C) was produced against peptide 163-179(pT175). For each ELISA set, phosphopeptides representative of other tau sites, as well as phosphorylated and unphosphorylated versions of the T169 and T175 containing peptides (Table 1), were used to investigate specificity within the tau protein. Further controls included a recombinant C-terminal fragment (244–441, numbered according to 2N4R tau) of 3R human tau, either non-phosphorylated or phosphorylated via GSK3β, as well as non-phosphorylated recombinant full-length 2N4R human tau. ELISA using PHF1 antibody, which recognizes tau phosphorylated at serines 396 (S396) and 404 (S404), was performed as a control (Fig 1D). As expected, PHF1 only positively recognized the GSK3β-phosphorylated 3R C-terminal tau fragment. The ELISA data for MHT1 indicates that this antibody is specific for the region of tau containing T169 and T175; however, it does not require phosphorylation at T169 and phosphorylation at T175 reduces the activity of the antibody. Furthermore, the antibody only recognizes the 163–179 peptides, suggesting that the proline-alanine-proline (PAP) motif that is specific to the 163–179 peptides is part of the recognition sequence for MHT1. The ELISA data for antibody MHT2 suggests that the antibody is similarly specific for the T169/T175 epitope, with the antibody again lacking phospho-specificity. In this case, however, it is phosphorylation at T169 as opposed to T175 that appears to hamper antibody activity. MHT2 strongly recognizes both 163–176 and 163–179 peptides but not the 165–176 peptide, suggesting that the N-terminal lysine-glycine (KG) motif in peptides 163–176 and 163–179 is critical for MHT2 recognition. Thus, both antibodies appear to recognize tau around the T169/T175 region, with neither antibody being phospho-specific.

### Antibodies MHT1 and MHT2 recognize tau by immunoblotting, but MHT1 demonstrates some non-specific activity

**Fig 2:**
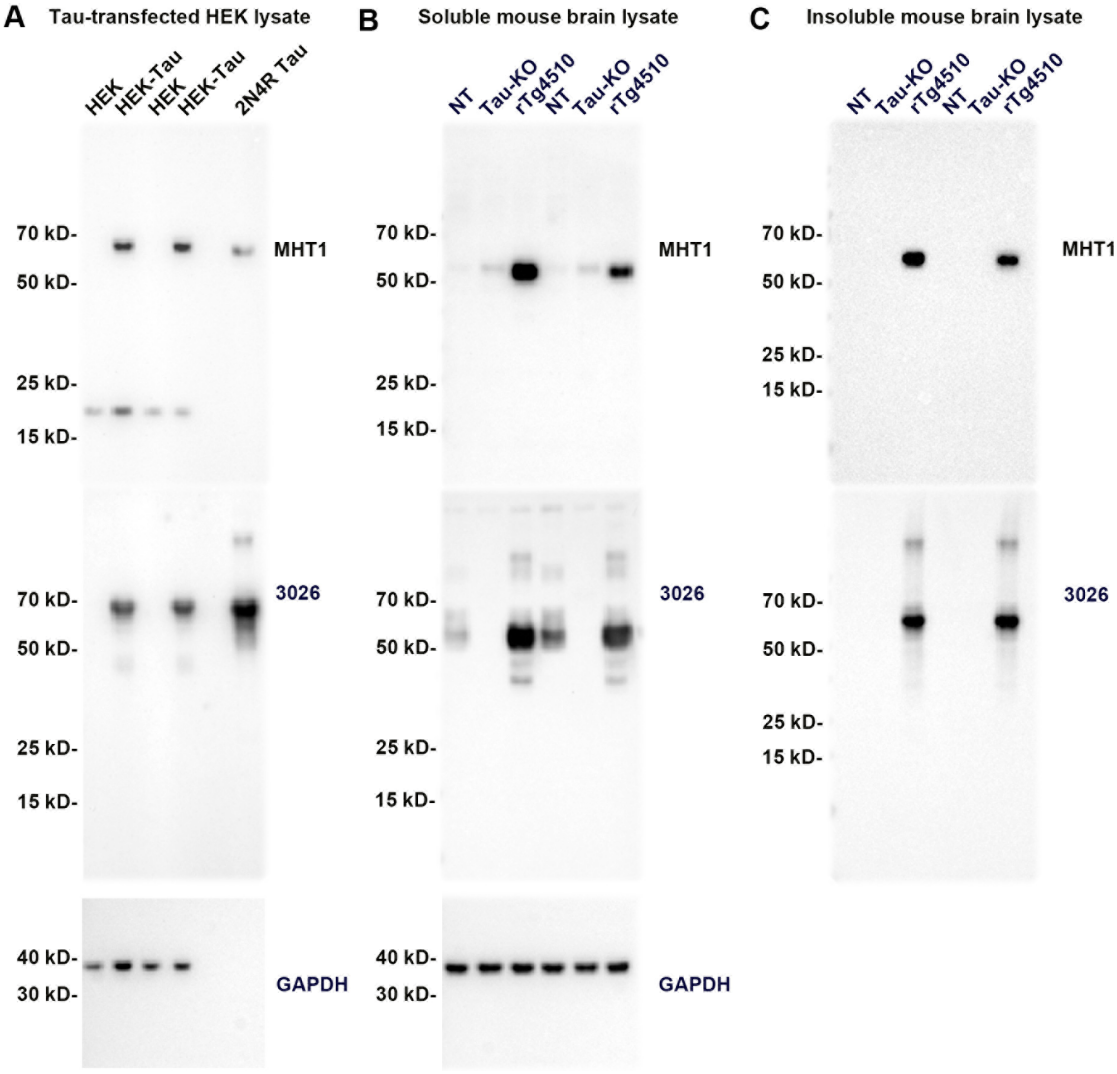
MHT1 antibody is not completely specific for tau in western blots of tau-transfected HEK cells and mouse brain homogenates. (A) Immunoblot of gels loaded with duplicates of tau-transfected HEK293T cell (HEK-tau) extracts as well as non-transfected (HEK) extracts and a single lane of 200 ng of recombinant 2N4R human tau (2N4R tau). The immunoblot was sequentially probed with tau antibodies MHT1 and 3026, and GAPDH. (B) Immunoblot of a gel loaded with soluble fractions of centrifuged brain homogenate. 6 month-old, female tissue from a nontransgenic mouse (NT), a tau knockout mouse (Tau-KO), and an rTg4510 mouse are loaded in duplicate. This immunoblot was also probed sequentially with antibodies MHT1, 3026, and GAPDH. (C) Immunoblot of a gel loaded with sarkosyl detergent insoluble fractions of the same fractionated tissue shown in B. In this case the immunoblot was only probed with antibodies MHT1 and 3026, as GAPDH is undetectable in the detergent-insoluble fraction. The mobilities of molecular mass markers are indicated on the left side of each blot.

**Fig 3:**
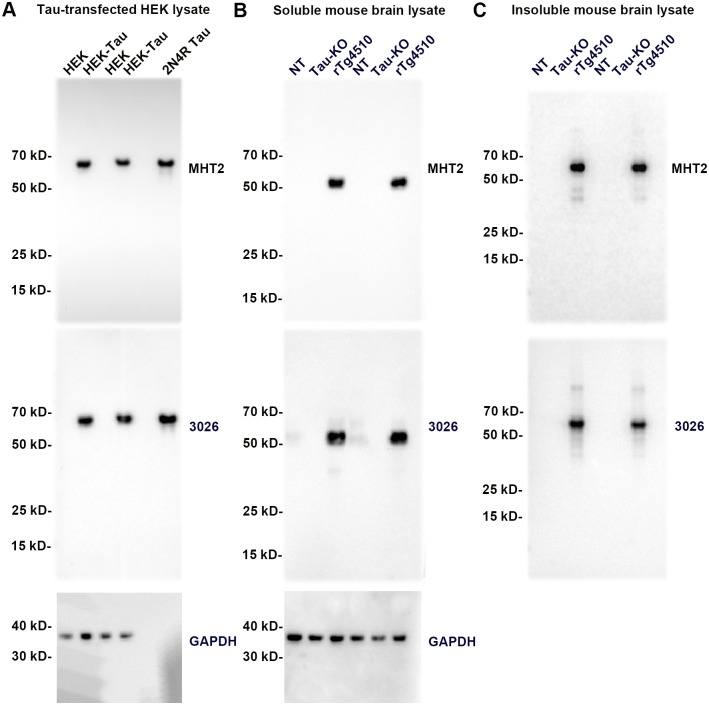
Antibody MHT2 specifically recognizes tau in western blots of tau-transfected HEK cells and mouse brain homogenates. (A) Immunoblot of gels loaded with duplicates of tau-transfected HEK293T cell (HEK-tau) extracts as well as non-transfected cell (HEK) extract and a single lane of 200 ng recombinant 2N4R human tau (2N4R tau). The immunoblot was sequentially probed with tau antibodies MHT2 and 3026, and a GAPDH antibody. (B) Immunoblot of a gel loaded with soluble fractions of fractionated brain homogenate. 6 month-old, female tissue from a nontransgenic mouse (NT), a tau knockout mouse (Tau-KO), and an rTg4510 mouse are loaded in duplicate. This immunoblot was probed with antibodies MHT2, 3026, and GAPDH. (C) Immunoblot of a gel loaded with sarkosyl detergent insoluble fractions from the same samples shown in B. In this case the immunoblot was only probed with MHT2 and 3026, as GAPDH is undetectable in the detergent-insoluble fraction. The mobility of molecular mass markers is indicated on the left of each blot.

Immunoblots were performed using antibodies MHT1 and MHT2 against whole-cell lysate of tau-transfected HEK293T cells (Figs 2A and 3A, respectively), as well as with soluble fractions (Figs 2B and 3B) and sarkosyl-extracted fractions representing aberrantly detergent-insoluble tau (Figs 2C and 3C) from rTg4510 transgenic, nontransgenic, and tau knockout mice brain tissue. For each blot, total tau antibody 3026 [11] was used to confirm the presence of tau, and glyceraldehyde 3-phosphate dehydrogenase (GAPDH) antibody GA1R was used as a loading control, with stripping of the blot performed between each sequential immunoprobe. MHT1 clearly recognizes tau in tau-transfected HEK293T cell lysate (Fig 2A), with total tau antibody 3026 confirming the presence of tau in the transfected HEK cells. Unfortunately, MHT1 also recognizes an unknown protein with a molecular weight of approximately 20 kD in both transfected and non-transfected cells, indicating it is not completely tau specific. In immunoblots of soluble and detergent-insoluble fractions of mouse brain homogenates, a lack of specificity of antibody MHT1 was also apparent. While MHT1 appears to recognize soluble tau in nontransgenic and rTg4510 mice (Fig 2B), as well as detergent-insoluble tau in rTg4510 mice (Fig 2C), the antibody also detects a protein of similar molecular weight to tau in the soluble fraction of tau-knockout mice (tau-KO) (16), which do not express murine tau. As this lack of specificity is clearly a problem for the utility of the MHT1 antibody moving forward, we did not proceed with further tests of MHT1 beyond these initial western blots.

MHT2 activity however, appears to be specific to tau. MHT2 cleanly recognizes tau in tau-transfected HEK293T cell lysate (Fig 3A), as well as in rTg4510 mice overexpressing human tau (Fig 3B) [14]. In the soluble fractions analyzed in Fig 3B, it is clear that MHT2 does not recognize anything in either nontransgenic mice or tau knockout mice, suggesting that MHT2 is not only specific for tau, but is further specific for human tau. Confirmation of endogenous murine tau in nontransgenic mice was shown with antibody 3026. Finally, MHT2 also recognizes detergent-insoluble tau from sarkosyl detergent-incubated brain tissue fractions (Fig 3C), further demonstrating tau-specificity. These data suggest that MHT2 is a robust and specific antibody for human tau; whereas, MHT1 is not altogether tau specific.

### Antibody MHT2 recognizes tau pathology in rTg4510 mice

**Fig 4:**
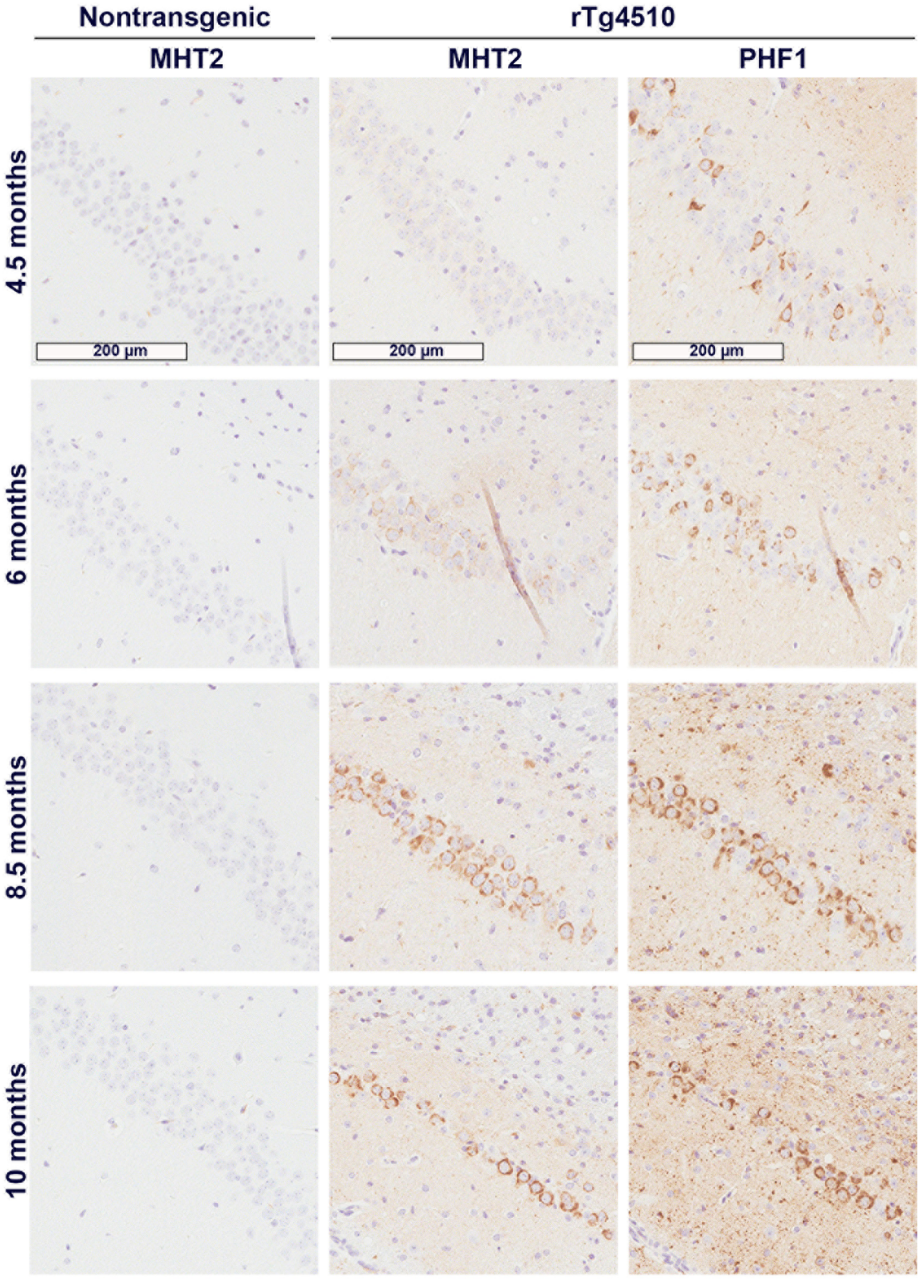
MHT2 antibody preferentially recognizes tau pathology in immunohistochemical staining of rTg4510 mouse tissue. Serial, sagittally sliced brain tissue from female rTg4510 mice was stained with MHT2 and PHF1 antibodies. Age-matched, nontransgenic tissue was stained with MHT2 as a control. Images depict 12x magnification of the CA1 region of stained tissue from representative samples at 4.5, 6, 8.5, and 10 months of age.

For further characterization of the antibody, MHT2 was used to analyze rTg4510 transgenic mouse brain tissue by immunohistochemistry (Fig 4). At all ages analyzed, MHT2 failed to immunostain nontransgenic brain; however, MHT2 clearly recognizes tau pathology in the CA1 of the hippocampus in rTg4510 mice starting with the 6-month age group, with signal robustness progressing with age beyond 6 months. The rTg4510 mouse model has been previously shown to develop pre-tangle pathology by 2.5 months of age and tangle pathology by 4.5 months (14). PHF1 immunostaining was used on transgenic mice as a control (Fig 4) to demonstrate that hyperphosphorylation of tau begins at a much earlier age point than MHT2 signal is detected. MHT2 appears to only recognize intracellular, cytoplasmic tau with very little background signal. It does appear that the antibody is either weaker or differentially specific as compared to PHF1, as it does not present the same robustness of staining, particularly in the earlier ages (4.5 and 6 months) of these mice. Because the ELISA data suggests that MHT2 does not readily recognize tau that is phosphorylated at T169, it is also possible that age-dependent changes in tau T169 phosphorylation affect MHT2 signal strength. Finally, it is also possible that a conformational preference may play a role in MHT2 activity, resulting in the smaller pool of tau pathology seen with MHT2 staining as compared to PHF1 staining.

### Antibody MHT2 recognizes tau pathology in human tauopathy cases

**Fig 5:**
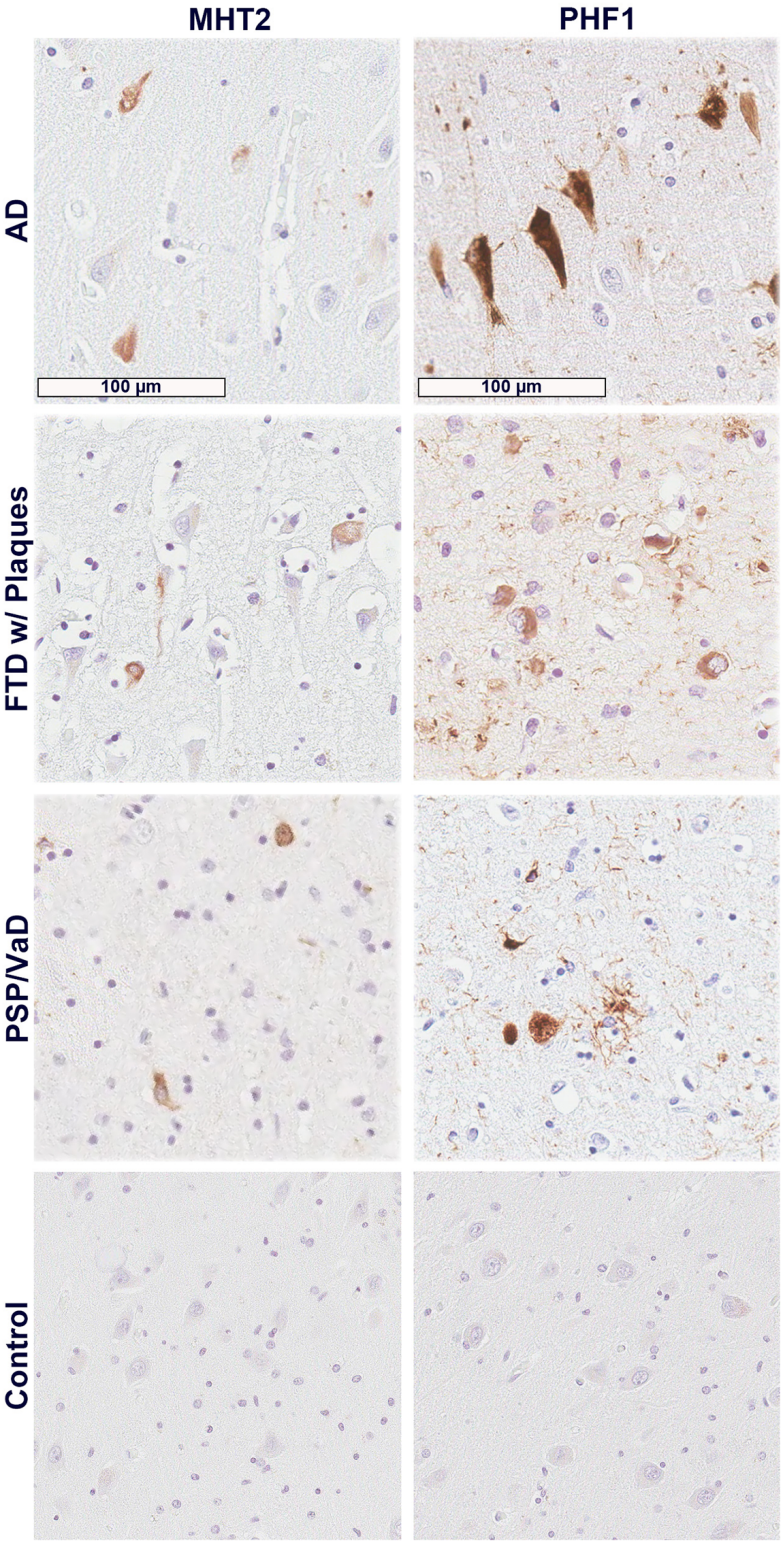
MHT2 antibody recognizes tau pathology in immunohistochemical staining of human cases of tauopathy. Tissue from the hippocampal region of an Alzheimer’s disease (AD) case, a frontotemporal disease (FTD) case with some amyloid plaques, and a progressive supranuclear palsy (PSP) case with concurrent vascular disease (VaD) are shown here stained with both MHT2 and PHF1 antibodies. Control, aged hippocampal tissue was also stained.

Antibody MHT2 was further used to stain human cases of tauopathy, with PHF1 again used as a positive control for hyperphosphorylated tau pathology. As with the rTg4510 mouse tissue staining, MHT2 again appears to be a less robust antibody compared to PHF1, as is demonstrated across hippocampal tissue from AD, FTD, and PSP cases (Fig 5). In these cases, however, MHT2 appears to be more specific for cytoplasmic neurofibrillary tangles (NFTs), as it does not appear to recognize specific pathological features seen by PHF1 in each case. PHF1 recognizes far more neuropil pathology in all three cases, as well as some plaques in AD and tufted astrocytes in PSP that MHT2 simply does not immunostain. Here again, MHT2 appears to be a highly specific tau antibody that preferentially recognizes fully developed NFTs in human tauopathies, much the same as in rTg4510-stained tissues.

### Sequencing and KD determination of MHT2

**Fig 6:**
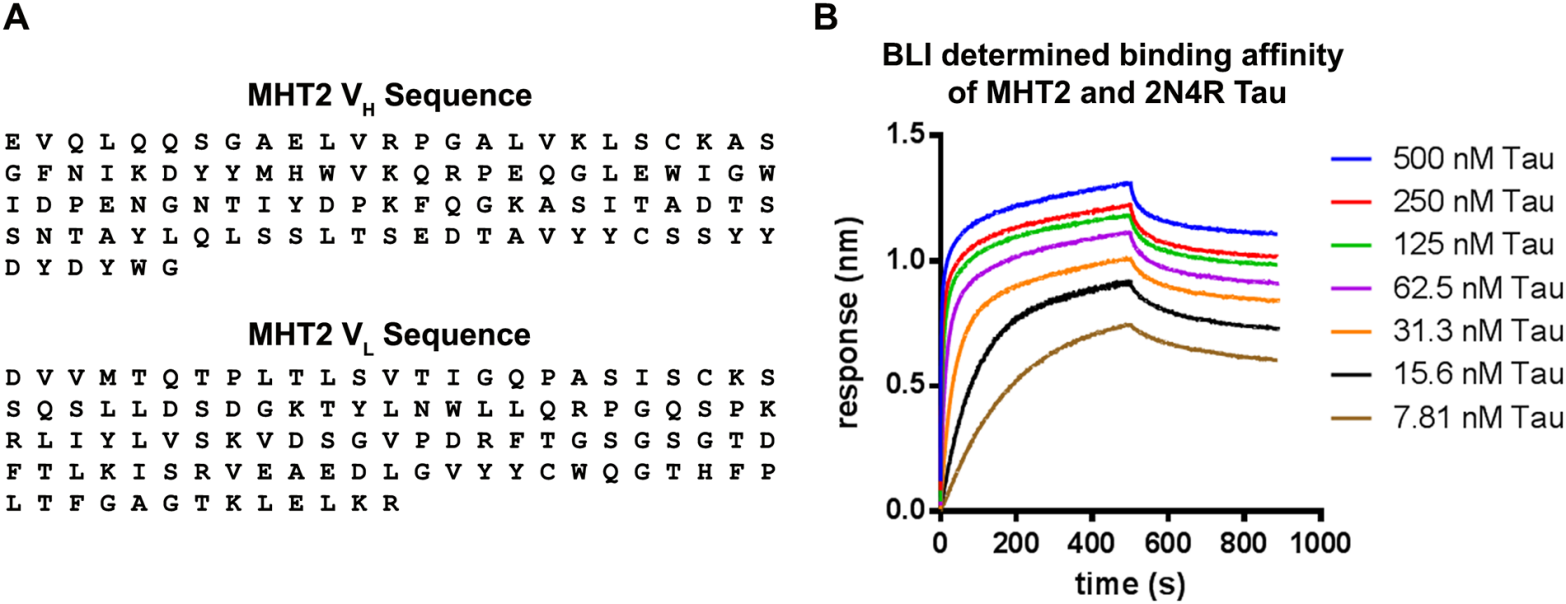
Variable chain sequences and 2N4R binding curve of MHT2. (A) Amino acid sequences of the variable heavy (V_h_) and light (V_L_) chains of antibody MHT2. (B) Binding curve for 2N4R human tau association to (0–500 seconds) and washout (500–1000 seconds) from biotinylated MHT2-bound streptavidin sensors. Various concentrations of 2N4R tau were used as shown in the key.

Having demonstrated MHT2 to be a highly specific tau antibody capable of recognizing NFTs, we took some final steps to more fully characterize the antibody. The isotype of MHT2 was determined to be IgG1. cDNA was generated from mRNA isolated from subcloned MHT2 hybridomas, and that cDNA was used to sequence the Variable heavy (V_H_) and Variable light (V_L_) chains of the MHT2 antibody. The amino acid sequences of the MHT2 variable regions are shown in Fig 6A. The equilibrium dissociation constant (KD) of antibody MHT2 and recombinant 2N4R human tau protein was also determined to be 6.39E-10 using bio-layer interferometry. The response curve for binding and washout of 2N4R tau on biotinylated MHT2-bound streptavidin sensors is shown in Fig 6B.

## Discussion

The main goal in approaching the antibody production and characterization described herein was to produce antibodies targeting tau epitopes that are phosphorylated by LRRK2, as demonstrated in our previous work examining LRRK2-mediated tau phosphorylation [3]. Our previous mass spectrometry analysis of *in vitro* G2019S LRRK2-phosphorylated tau indicated that T169 and T175 of tau are major targets for LRRK2-mediated phosphorylation. As such, we designed phosphorylated peptides corresponding to the pT169 and pT175 epitopes on tau, which were used as antigens in monoclonal antibody production.

Peptides comprised of residues 163–176 and 165–176 of tau (Fig 1A and Table 1), wherein T169 was phosphorylated, were first used as antigens in unsuccessful attempts at monoclonal antibody production. Thus, we expanded the size of the peptide to 163–179, also shown in Fig 1. We immunized mice with two variants of this longer peptide, one phosphorylated at the T169 site [163-179(pT169)] and the other phosphorylated at T175 site [163-179(pT175)]. Hybridomas generated from mice immunized with these peptides appear to be specific for the T169/175 tau epitope, as assessed by ELISA (Fig 1B and 1C). While the ELISA data suggests that the MHT1 and MHT2 antibodies specifically target the tau epitope represented by the 163-179(pT169) and 163-179(pT175) antigens, neither antibody appears to specifically recognize tau phosphorylation at those sites. The reduction in activity of MHT1 and MHT2 when T175 or T169 are phosphorylated, respectively, suggests that each of those sites is important for the recognition of tau by the respective antibody. Owing to the apparent difficulty in producing phosphorylation-specific antibodies targeting these tau epitopes, as well as the relative novelty of tau antibodies targeting these epitopes, we proceeded with further characterization of these antibodies.

Western blot analysis of MHT1 (Fig 2) revealed that the antibody is not entirely tau specific, as it appears to recognize a 20 kD protein in HEK293T cells as well as an approximately 55 kD protein that appears to be upregulated in tau knockout mice, as compared to nontransgenic and rTg4510 mice. This unexpected promiscuity of the MHT1 antibody, exacerbated by the similarity in size between tau and the unidentified 55 kD protein, resulted in our cessation of further characterization of the MHT1 antibody. These results do however emphasize the importance of utilizing proper negative controls in the characterization of antibodies.

Western blot analysis of MHT2 (Fig 3) provided more promising results in terms of generating a highly tau-specific antibody. MHT2 appears to recognize only tau protein, with distinct single bands presenting in the HEK293T and soluble brain lysate westerns. Multiple weaker bands are present in the insoluble brain lysate westerns, but those bands are likely representative of polymerized tau species as well as degradation products. A further point of interest stands out in the soluble brain lysate western, as MHT2 does not appear to recognize any tau in nontransgenic mice. The murine tau expression level in nontransgenic mice should be far lower than that of the overexpressed human tau in the rTg4510 mice, but the 3026 total tau antibody re-probe of the soluble brain lysate blot does indeed confirm the presence of tau in the nontransgenic mice. Taken together, this information suggests that MHT2 recognizes human tau but not murine tau. The differences between human and murine tau at amino acids 165 and 166 (Fig 1A), which are contained within the antigen used to generate MHT2, support the plausibility of this finding. We concluded that antibody MHT2 appears to specifically recognize human tau around the T169 epitope leading us to further characterize the antibody via immunohistochemistry.

The use of the MHT2 antibody to probe a mouse model of human tauopathy (Fig 4) by immunohistochemistry provided interesting results. The rTg4510 tissue immunostaining (Fig 4) shows pathology that appears to be cytoplasmic, tau NFTs. With respect to animal age, the intensity and quantity of MHT2 staining lags behind that of PHF1 antibody, as well as immunostaining with other phospho-tau antibodies previously performed on rTg4510 tissue [14]. PHF1, which recognizes phosphorylated S396 and S404 of tau, is used here as a positive control for tau pathology. The MHT2 signal is only readily apparent starting with the 6-month tissue, and only appears to recognize NFTs to a similar degree as PHF1 starting at 8.5 months. This could be the result of a progressive, age-dependent decrease in phosphorylation at T169, as T169 phosphorylation appears to mitigate MHT2 activity as seen by ELISA (Fig 1C). Alternatively, the MHT2-targeted epitope may simply be conformationally unavailable under normal physiological conditions, yet accessible in later-stage tau pathology, hinting at a conformational specificity to this antibody. Additionally, MHT2 appears to recognize only somatic, cytoplasmic tau, while PHF1 recognizes pathology in neurites as well. Taken together, the MHT2 data in Fig 4 suggests that this antibody recognizes fully developed, somatic NFTs in the rTg4510 mouse model of tauopathy.

A similar pattern of staining is seen in immunohistochemical probes of human cases of tauopathy using MHT2 (Fig 5). MHT2 again appears to recognize late stage tauopathy in the form of somatic NFTs, and does not show an affinity for tau containing plaques, neurites, or astrocytic tufts that PHF1 recognizes. This again suggests that there is a qualitative difference in the presentation of the MHT2 epitope and PHF1 epitopes in normal and disease-related tau species.

Overall, we show here a novel, monoclonal tau antibody that cleanly detects mature NFT tau pathology in transgenic mouse tissue as well is in human cases of tau-related diseases. While the antibody is not phospho-specific, this human-specific, monoclonal antibody targeting the region around T169 in tau appears to be unique in the literature. Given this novelty as well as the antibody’s distinct affinity for somatic NFTs, it may prove useful in further analysis of tau and tau based diseases.
